# Muscle irisin response to aerobic vs HIIT in overweight female adolescents

**DOI:** 10.1186/s13098-017-0302-5

**Published:** 2017-12-28

**Authors:** Carolina Archundia-Herrera, Maciste Macias-Cervantes, Bernardo Ruiz-Muñoz, Katya Vargas-Ortiz, Carlos Kornhauser, Victoriano Perez-Vazquez

**Affiliations:** Department of Medical Science, Division of Health Science, University of Guanajuato, 20 de enero 929. Col. Obregon, CP 37250 Leon, Guanajuato Mexico

**Keywords:** Exercise, Irisin, Obesity, Skeletal muscle biopsy

## Abstract

**Background:**

Exercise stimulates the production of fibronectin type III domain-containing protein 5 (FNDC5), which is cleaved to release a protein called irisin. This protein induces browning of white adipose tissue resulting in increased thermogenesis. Different studies have measured circulating irisin at baseline and in response to exercise among a wide variety of individuals; yet, regarding the effect of different exercise intensities in obese adolescent girls, limited insight is available. This study compares the effect of acute aerobic exercise of moderate intensity and high-intensity interval training (HIIT) on irisin levels in skeletal muscle and plasma of sedentary overweight or obese female adolescents.

**Methods:**

The aerobic group (n = 15) and HIIT group (n = 15) underwent anthropometric and metabolic measurements, electrocardiogram, peak oxygen uptake (VO_2peak_), and two vastus lateralis muscle biopsies before and after session of workout. The session of aerobic exercise included cycling at 65% of their peak heart rate (HRpeak) for 40 min. In the HIIT group, exercise included six bouts of 1 min at 85–95% HRpeak separated by 1 min of recovery. Irisin levels were evaluated in samples of skeletal muscle (western blot) and plasma (ELISA).

**Results:**

The levels of expression of irisin in skeletal muscle increased significantly after a session of HIIT (p < 0.05), while aerobic exercise no affect irisin levels. No significant differences between the groups in plasma irisin levels were found.

**Conclusions:**

The increase in muscle irisin levels was observed only following HIIT session. No increases in plasma irisin concentration were observed.

## Background

There is considerable evidence that overweight and obesity are serious worldwide public health problems which are shadowed by an increased risk of developing non communicable diseases such as, type II diabetes mellitus (DM2) and cardiovascular diseases [1, 2], which affect both developed and developing countries triggering a robust burden for the health systems in the upcoming decades [3].

Physical activity reduces the risk of cardiovascular events, DM2, hypertension, colon cancer, breast cancer and, depression [4, 5].

The skeletal muscle is considered an excretory organ with the ability to communicate with other tissues/organs. Many proteins produced by skeletal muscle depend on contraction. Therefore, physical inactivity, probably leads to altered muscle response [6]. Irisin, a peroxisome proliferator-activated receptor gamma coactivator 1-alpha (PGC-1α) dependent myosin was recently described [7]. Muscle contraction increases PGC-1α when exercising which in turn increases the expression of FNDC5. This protein is cleaved to release irisin which increases in response to exercise induced browning of white adipose tissue resulting in increased thermogenesis and has been identified in muscle and plasma of mice and humans [7].

However, a gap in the literature exists regarding the understanding of different types of exercise intensities on levels of irisin. It has been reported that long sessions of moderate intensity exercise (> 1 h at 65% VO_2max_) increase muscle oxidative capacity and improve both physical fitness and central adiposity in adolescents with obesity [8, 9]. Hight-intensity interval training (HIIT), another type of exercise, causes adaptations that resemble traditional aerobic training despite a substantial reduction in the total time commitment and exercise volume [10]. HIIT is a potent stimulus for improving several important metabolic and cardiovascular risk factors in men and women between 40 and 75 years with type 2 diabetes [11]. Furthermore, changes on irisin concentrations in response to exercise contradictory results has been reported. It has been reported that irisin levels increased significantly after one session of HIIT, while pilates has not affect in overweight woman [12]. However, in healthy males changes were not found on mRNA irisin in skeletal muscle after an acute bout of HIIT, although after 20 days of training, mRNA irisin levels increased [13]. Conversely, sprint training for 4 weeks significantly decreased the resting serum irisin concentration in healthy males [14]. Recently, Fox et al., using a single and multiple meta-regressions, suggested that an acute bout of exercise was accompanied by a post-exercise average increase in irisin concentration of 15% [15]. Therefore, the objective of this study was to compare the effect of one bout of aerobic exercise of moderate intensity and HIIT exercise on irisin levels in skeletal muscle and plasma of sedentary adolescent girls who were overweight or obese.

## Methods

A cross-sectional controlled trial was done. Thirty female adolescents were included from public schools in León, Guanajuato, México. Data were collected in 2013–2014 and analyzed in 2014.

### Subjects

Thirty female adolescents were randomized to aerobic (n = 15) or HIIT (n = 15) group. The inclusion criteria were: sedentary (90 min or less of exercise/week in the last 2 months), between 14 and 18 years old, with overweight or obesity [overweight = body mass index (BMI) greater than one standard deviation for age and sex, obesity = BMI two standard deviations higher for age and sex] [16], without muscle alterations, on-going nutritional or drug treatments that could affect their weight and deprived of consumption of alcohol or drugs.

Conform to the principles outlined in Declaration of Helsinki 2013, the Ethics Committee of the Medical Sciences Department at the University of Guanajuato approved this investigation. Written informed consent was obtained from the parents and participants.

### Exercise session

#### Aerobic group

The participants started with 5 min of warming in the cycle ergometer and were asked to reach a 65% HRpeak. Once they reached it, they were asked to keep it for 40 min. When finished, they had a 5-min period of cooling down.

#### HIIT group

HIIT session began with 5 min of warming up, subsequently subjects performed six bouts of 1 min at 85–95% HRpeak (obtained during baseline VO_2peak_ test) separated by 1 min of recovery at easy intensity. The experimental setting was based on a previous study [11, 17, 18].

The week before to the exercise session the all teenagers performed an exercise stress test on a cycle ergometer (Monark, Ergomedic 828 E, Varberg, Sweden) with the Taguchi modified protocol [19]. Briefly, the test consisted of a period of familiarization with rhythmic pedaling for a 5-min warm up period. The ergometer was calibrated, then, the participants started to rhythmically pedal at 60 rpm against a load that began with 1.5 kp for 2 min and it gradually increased every 2 min 0.5 kp. The test consisted of multistage incremental effort with progressive increase of the load in each stage. Both, the basal heart rate and HR_peak_ were recorded every minute with a heart rate monitor (Polar RS400SD, Kempele, Finland). The test ended when they reached the exhaustion or if the participants did not keep with the cadence of pedaling. The leg-ergometer equation was used to estimate the peak oxygen consumption (VO_2peak_) [20]. VO_2peak_ (ml/kg/min) = 1.8 [work rate (kg m/min)/body mass (kg)] + 7. VO_2peak_ was used as an index of cardiorespiratory fitness. To rule out contraindications for exercising, a 12-lead electrocardiogram (ECG) was obtained (Combo Resting 12-Lead ECG. 4.0 Premier, DM Software, Stateline, USA).

### Biopsies

Two biopsies of the vastus lateralis muscle in the dominant leg were taken. The first biopsy was performed 2 days before the exercise session (baseline) and the second biopsy was performed 30 min after exercise session, ~ 2 cm proximal to the pre-exercise session site. The participants refrained from exercising 2 days before each biopsy. In aseptic conditions, the subcutaneous juxta-aponeurotic cellular tissue was infiltrated with 6 ml of Xylocaine 1%. A 2–3 mm incision was performed with a number 22-scalpel blade, with ultrasound guidance. A biopsy needle of 14 g in diameter and 11 cm in length (Tenmo T1411) was introduced to reach the muscle mass, obtaining 100 mg approximately. The tissue obtained was washed with a buffer (20 mM Tris/HCl pH7.8, 10 mM EDTA, 2 mM DTT and protease inhibitor) and stored at − 80 °C for later analysis by Western blot.

### Primary outcome measures

The measures were the levels of irisin in muscle and plasma. These were obtained by Western blot and Elisa analysis respectively.

The content of irisin before and after aerobic or HIIT session was determined in skeletal muscle by western blotting using triplicate samples of muscle tissue. Briefly, the protein was extracted with Sample Grinding Kit (GE Healthcare, Switzerland) and centrifuged at 16,000 rpm/10 min. The supernatant was dissolved in a sample buffer (0.5 M Tris–HCl pH6.8, 25% glycerol and 2% SDS). The protein concentration was determined by the method of Lowry [21]. The standard curve was performed in triplicate. Absorbance was read in spectrophotometer (Multiskan GO, Thermo Scientific, Finland) to 750 nm.

Denaturing polyacrylamide gels (SDS-PAGE) were prepared at 12% with 4% stacking gel leaving it to polymerise for 40 and 20 min respectively. Twenty μg of protein premixed with β-mercaptoethanol (25%) and loading buffer (63 mm Tris–HCl, 2% SDS, glycerol and bromophenol blue 0.0025%) were denatured at 96 °C in a water bath for 5 min; then they were separated on an electrophoresis chamber (Mini Protean Tetra Cell, Biorad, Mexico) at 120 V for the necessary time to reach 1 mm before the end of the gel. The transfer of proteins from gel to PVDF membranes was performed in a humid chamber transfer (Trans-Blot SD Semi-Dry Transfer Cell, Bio-Rad) for 1 h at 90 V. Once the proteins were transferred to PVDF membranes, they were blocked with TBST buffer (Tris 20 mM, NaCl 500 mM, Tween 20 at 0.05%, pH7.5) and 4% skim milk overnight then incubated for 3 h with primary antibodies Anti-FNDC5 (1:2000) (EPR12209) (ab174833) (Abcam, USA) and α-tubulin (1:2000) (ab15246) (Abcam, USA) as a loading control. They were incubated for 2 h with the secondary antibody IgG-HRP (1:6000) (ab6721) (Abcam, USA). At the end of each stage, the membranes were washed 3 times with TTBS. The proteins in the membrane were detected by using the chemiluminescence Kit Wester LightningTM Plus-ECL (Perkin Elmer. INC, USA). Finally, densitometry analyses were conducted using Image Laboratory Software (Biorad, Mexico) and results were normalised according α-Tubulin values.

### Blood sampling and analysis

Two samples (12 ml each one) of peripheral venous blood were obtained using a vacutainer system. To the first baseline sample, the participants reported to the laboratory at 8:00 h following an overnight fast and 48-h abstention from vigorous physical activity. The second sample was obtained 30 min after finishing the exercise session. Blood samples were treated to measure plasma irisin by ELISA with the Irisin kit (Cat.# EK-067-29 (Phoenix Pharmaceuticals, Burlingame, CA, USA), with a coefficient of variations (CVs) for intra assay: 5–7%; CVs for inter-assay: 12–15%; detection limit range: 0.1–1000 ng/ml. The assays used to detection of irisin were previously validated.

Blood samples were also treated to measure secondary outcomes such as glucose, insulin, and lipid profile. Glucose was determined using the enzymatic colorimetric method: glucose oxidase/peroxidase (BioSytems, USA). Insulin levels were determined by radioimmunoassay (Human Insulin Specific, MILLIPORE. Darmstadt, Germany). Lipid profile was determined using the enzymatic colorimetric methods CHOD-POD and GPO-POD (SPINREACT, Spain). Index of insulin resistance (HOMA-IR) was calculated according to Matthews et al. [22].

### Other secondary outcomes were the anthropometric measurements

The basic measurements, height, and weight were recorded with an accuracy of 0.5 cm and 0.1 kg respectively (Seca 813, Hamburg, Germany). Participants underwent an anthropometric profile performed by a certified person on the international standards for anthropometric assessment of the International Society for the Advancement of the Kinanthropometry (ISAK). Measurements were performed in duplicate to decrease the assessment error. For the optimal requirement for evaluation, participants were asked to fast (> 8 h), minimum rest of 8 h, to present themselves neat and hydrated, and to wear light clothing.

All measurements were performed at the University of Guanajuato, Medical Sciences Department.

### Statistical analysis

Sample size was determined as n = 30 since the irisin variance is not known, the power calculation was performed at posteriori resulting a power of 96%. The Kolmogorov–Smirnov and Shapiro–Wilk test were used to determine the distribution of the variables. The effect of time and type of exercise was analyzed by repeated measures ANOVA. Significance was considered at p < 0.05. Statistical analysis was performed with the software Statistica (StatSoft V6, Tulsa, OK, USA).

## Results

The groups were homogeneous at baseline, the descriptive characteristics of participants of the aerobic and HIIT group are shown in Table 1. According inclusion criteria, participants were overweight or obese teenagers with low cardiorespiratory fitness. Table 2 shows the metabolic variables of the aerobic group and the HIIT group pre- and post-exercise session. No significant changes were observed.

**Table 1:** Descriptive and anthropometric initial characteristics

Descriptive characteristics			
Variables	HIIT (n = 15)	Aerobic (n = 15)	p
Age (years)	16.13 ± 1.64	15.47 ± 1.73	0.2877
Weight (kg)	82.62 ± 14.09	84.21 ± 16.88	0.781
Height (m)	1.61 ± 0.06	1.6 ± 0.07	0.6539
BMI (kg/m^2^)	31.65 ± 4.83	32.67 ± 5.91	0.6079
Percentile of BMI	92.67 ± 4.17	94 ± 2.07	0.2767
Waist circumference (cm)	93.46 ± 9.9	95.46 ± 15.51	0.6769
Fat mass (%)	34.39 ± 2.32	40.5 ± 17.92	0.1997
Muscle mass (%)	29.91 ± 3.14	41.79 ± 25.48	0.0833
SBP (mmHg)	113.67 ± 10.42	110.57 ± 7.17	0.3629
DBP (mmHg)	70.6 ± 8.36	71 ± 6.16	0.8852
VO_2peak_ (ml/kg min)	28.23 ± 3.96	27.11 ± 4.14	0.454

Data are expressed as mean ± SD

*BMI* body mass index, *SBP* systolic blood pressure, *DBP* diastolic blood pressure, *VO*_*2peak*_ peak oxygen consumption

p values were obtained by means of *t test*

**Table 2:** Metabolic variables of aerobic and HIIT groups pre and post exercise session

Training groups					
Variables	HIIT (n = 15)		Aerobic (n = 15)		
	Pre exercise	Post exercise	Pre exercise	Post exercise	p
Fasting glucose (mg/dl)	84.45 ± 4.42	83.31 ± 6.11	89.67 ± 11.94	88.24 ± 8.57	0.71
Fasting insulin (μUI/ml)	14.86 ± 6.84	15.04 ± 6.94	13.87 ± 8.13	12.57 ± 7.12	0.19
HOMA-IR	3.13 ± 1.53	3.11 ± 1.54	3.18 ± 2.33	2.74 ± 1.53	0.28
Total cholesterol (mg/dl)	159.55 ± 27.14	162.75 ± 26.29	144.53 ± 19.8	141.21 ± 20.47	0.65
HDL cholesterol (mg/dl)	58.33 ± 7.39	59.43 ± 6.66	60.43 ± 8.9	58.78 ± 7.71	0.59
LDL cholesterol (mg/dl)	67.95 ± 26.01	71.43 ± 25.61	59.71 ± 15.2	60.77 ± 21.63	0.87
Triglycerides (mg/dl)	166.57 ± 93.16	159.71 ± 108.08	122.19 ± 40.49	108.46 ± 33.63	0.32

Data expressed as mean ± SD

*HDL* high-density lipoprotein, *LDL* low-density lipoprotein, *VLDL* very low-density lipoproteins

p value was obtained by analysis of repeated measures ANOVA

**Fig. 1:**
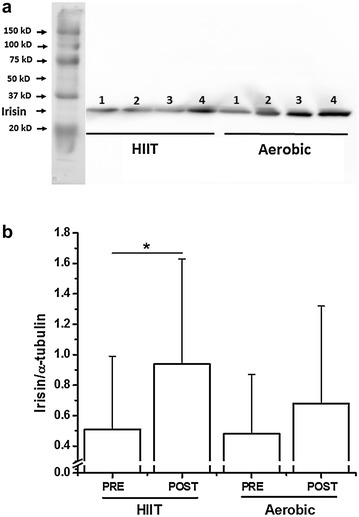
Effect of HIIT or aerobic exercise session on irisin levels in muscle. **a** Representative western blot of irisin in muscle. **b** Densitometry analysis of the irisin/α-tubulin ratio. 1 and 3, pre-HIIT or aerobic session; and 2 and 4, post-HIIT or aerobic session. Data are expressed in mean ± SEM (n = 15) *p < 0.05

We observed one band at approximately 24 kD corresponding to irisin. There was a significant interaction (p < 0.05) between groups. The levels of content of muscle irisin/tubulin pre- and post-HIIT session increased significantly (0.51 ± 0.48 to 0.94 ± 0.69, p < 0.05), while the content of irisin did not change after aerobic session (0.48 ± 0.39 to 0.68 ± 0.64, p = 0.3 (Fig. 1). No significant changes were observed in the plasma irisin concentrations pre- and post-exercise session (Table 3).

**Table 3:** Expression of plasma irisin pre and post exercise session

	HIIT (n = 15)		Aerobic (n = 15)		
	Pre exercise	Post exercise	Pre exercise	Post exercise	p
Plasma irisin (ng/ml)	3.59 ± 1.29	3.70 ± 1.26	3.66 ± 0.80	3.56 ± 0.69	0.62

Data expressed as mean ± SD

p value was obtained by analysis of repeated measures ANOVA

## Discussion

This study was designed to test the effect of different modalities of exercise on irisin levels in skeletal muscle and plasma after one bout of exercise. Our hypothesis was that the irisin levels in muscle and plasma would increase after one session of exercise and, that this increase would present different acute responses in both training groups, indeed, the results obtained in this research confirm a significant increase in the levels of irisin in muscle after a session HIIT exercise.

We found increased levels of irisin of skeletal muscle same as reported by several authors [23–25]. In accordance with the results of Tsuchiya et al., who also observed increased levels of irisin in the group of high-intensity exercise when compared with the low-intensity group [26] we found a significant difference between groups. However, in other investigations no changes have been found in the expression of irisin (mRNA or protein) [27, 28], the difference could be because in these investigations the exercise was performed at long term. In the investigation of Pekkala et al. different exercise protocols were implemented, such as acute load and long-term training and using different intensities, the increase in the expression of irisin in skeletal muscle was only confirmed with acute exercise at high intensity [28]. The individual response to exercise is highly variable followed by several training protocols [29]; the results so far analyzed, could explain that the increase in the expression of irisin in skeletal muscle depends on the intensity of the acute load of exercise.

We did not find an increase in the plasma concentration of irisin after a single bout of either aerobic exercise or HIIT. Consistent with our results, different studies, have failed to report an increase of irisin in plasma concentrations [27, 28, 30–32], while others have reported a modest increase during moderate and high intensity exercise [1, 33]. These studies were done in different populations with a large spectrum of age, BMI, and physical fitness, and using a variety of exercise modalities such as swimming, cycling or treadmill, partially explaining the results discrepancies [34]. However, the studies where there was an increase in the plasma concentration of irisin, the determination was made with blood samples taken immediately after the end of the exercise session [33, 35]. Furthermore, the use of different ELISA kits and their validity have been questioned explaining partially, the discrepancies in the reported results. Aviscera Biosciences, Santa Clara, CA (USA), and USCN Life Science, Wuhan (China) are two ELISA kits that have been used in different studies and have an inaccuracy greater than 8%. The ELISA kits used in this study was the EK-0670-29 Phoenix Pharmaceuticals, Burlingame, CA (USA), which has an intra-assay inaccuracy of 7% and has been validated against the gold standard [36]. Indeed, our results are like those recently reported by Jedrychowski [37], who used tandem mass spectrometry in sedentary young healthy participants (n = 6 males, 25 ± 5 years, BMI = 24.3 ± 2.5 kg/m^2^) following 12 weeks of high-intensity aerobic training, showing that irisin concentrations are present at 3.6 ng/ml in sedentary individuals and are significantly increased to 4.3 ng/ml in individuals undergoing aerobic interval training.

Research related to irisin expression and exercise is complex. To start elucidating how irisin response to exercise, different issues must be address. First, irisin source, Moreno-Navarrete et al., found that muscular levels of irisin was 200 times higher in relation to adipose tissue [38]. Approximately 72% irisin comes from the muscle tissue and the remaining 28% comes from adipose tissue therefore skeletal muscle is the main source of plasma irisin [39]. In this respect, one of the main strengths of this study is that was performed directly on muscle skeletal biopsies and provides information about pure pediatric physiology.

Second, muscle mass and aerobic capacity hold an important role. Some studies have reported that both are involved in the regulation of plasma concentrations of irisin [35, 40]. It has been described a negative association between VO_2max_ and irisin levels in active subjects, even more, sedentary subjects in the same age group as active subjects have higher irisin levels when compared [35]. Huh et al. found that acute exercise increases circulating irisin concentrations while chronic exercise produces no change, or even decreases concentrations [23, 41]. Higher values of VO_2max_ in active subjects might indicate a better cardiovascular condition and therefore a lesser muscle feedback [42], implicating an adaptive response to increased muscular capacity. A similar phenomenon has been reported with other cytokines such as IL-6 [43]. It could be attributed to irisin a similar kinetic to that of IL-6, which increases immediately after exercise to regulate thermogenesis and metabolism, although it is negatively correlated with long-term metabolic variables [44].

Third, irisin response time to exercise; Initial studies reported that circulating levels of irisin increased 30 min after exercise [23] in conjunction with an immediate increase after high-intensity interval exercise, continuous moderate-intensity exercise, and resistance exercise declining 1 h later [45]. Based on these results, the design of the present study was developed. However, our study did not show changes in the plasma concentrations of irisin 30 min post-exercise. It is possible that irisin concentrations transiently increase during exercise, but decrease during recovery time. A recent study measured irisin levels during and after exercise which helped characterized irisin behavior because of moderate and high intensity exercise. They reported a modest increase during exercise which continued for 125 min for moderate exercise but returned to the baseline after 15 min for high intensity exercise [33]. Recently it has been postulated that at the beginning of exercise the concentration of irisin increases suddenly, reaching a peak at 45 min, decreasing subsequently (90 min) [35], therefore it is possible that in the present study the concentration of irisin might have increased in the initial stage of the exercise to subsequently decrease in the recovery stage. One of the limitations of this study is that plasma irisin was not determined during the exercise, nor immediately after the end of it. While another limitation was that energy expenditure was not adjusted in both exercise groups and this could be different. Future research is necessary to understand the molecular and metabolic mechanisms underlying the transient increase in irisin during exercise.

Recent studies with primary cells from human skeletal muscle (HSMC) treated with recombinant irisin, have shown that irisin facilitates the use of glucose and fatty acids uptake by regulating the levels of ATP throughout an effect of “saving” glycogen [35]. Exercise induces transcription of FNDC5 prolonging the effect of irisin in the muscle so that irisin functions as a signal that facilitates the metabolism, independent of the effects on the browning of adipocytes [41]. On the other hand, muscle production and secretion of irisin is also mediated by SMAD3 (mothers against decapentaplegic homolog 3), molecule that modulates energy metabolism and regulate body weight. The SMAD3 protein in response to exercise is regulated differently in obese mice than in lean mice [46]. These studies partially explain the role of irisin and its time of action at a muscle level but these finding warrant further study and remain speculative.

## Conclusions

In conclusion, the findings of this study demonstrate that a bout of HIIT exercise increases acutely irisin levels in skeletal muscle without changes in plasma irisin levels. However, aerobic exercise no affected circulating nor muscle irisin levels.

## Abbreviations

FNDC5: fibronectin type III domain-containing protein 5
HIIT: high-intensity interval training
VO_2peak_: peak oxygen consumption
DM2: type II diabetes mellitus
PGC-1α: peroxisome proliferator-activated receptor gamma coactivator 1-alpha
BMI: body mass index
SBP: systolic blood pressure
DBP: diastolic blood pressure
HDL: high-density lipoprotein
LDL: low-density lipoprotein
VLDL: very low-density lipoproteins
SMAD3: mothers against decapentaplegic homolog 3
